# Healing of Oral Lichenoid Lesions following Replacement of Dental Amalgam Restorations with Feldspathic Ceramic Inlay-Onlay Restorations: Clinical Results of a Follow-Up Period Varied from Three Months up to Five Years

**DOI:** 10.1155/2018/7918781

**Published:** 2018-12-03

**Authors:** Burcin Karatasli, Gokcen Karatasli, Ozgur Mete, Mehmet Ali Erdem, Abdulkadir Burak Cankaya

**Affiliations:** ^1^DDS, Ph.D., Department of Prosthodontics, Faculty of Dentistry, Istanbul University, Capa, Istanbul, Turkey; ^2^Assoc. Prof., Department of Oral and Maxillofacial Surgery, Faculty of Dentistry, Istanbul University, Capa, Istanbul, Turkey; ^3^DDS, Ph.D., Program of Oral and Dental Health, Vocational School of Higher Education, Beykent University, Beylikduzu, Istanbul, Turkey; ^4^MD, FRCPC, Associate Professor, Department of Pathology, University Health Network, Toronto, ON, Canada; ^5^Department of Laboratory Medicine and Pathobiology, University of Toronto, 1 King's College Circle, Toronto, ON, Canada

## Abstract

**Objective:**

Previous studies have shown the effect of amalgam removal on the healing of oral lichenoid lesions (OLLs); however, no specific replacement materials have been suggested. The present series evaluated long-term results following the complete replacement of amalgam restorations with feldspathic ceramic inlay-onlay restorations for a group of patients with OLLs whose lesions were suspected to be related to amalgam restorations.

**Materials and Methods:**

Twenty-four patients who had OLLs suspected to be related to their amalgam restorations were initially recruited. The patients underwent patch tests for a series of dental materials, in addition to clinical and histopathological examination. Sixteen (67%) of the 24 patients had their amalgam replaced with feldspathic ceramic inlay-onlay restorations and were examined within a follow-up period of 3 months to 5 years.

**Results:**

After 3 months of clinical follow-up, complete healing (63%) was noted in all patients with OLLs whose lesions were in only close contact with their amalgam restorations. Healing was significantly related to the combination of lesions with close contact with the amalgam restoration and a diagnosis of OLL (x^2^ test, P=0.02).

**Conclusion:**

Feldspathic ceramic can be safely used as a replacement material for patients with OLLs to diminish adverse reactions to amalgam restorations.

## 1. Introduction 

Various components found in amalgam often result in hypersensitivity reactions [1–3]. It is evident from many previous studies that certain components of dental amalgam restorations may induce the formation of oral lichenoid lesions (OLLs) [4–6]. In the absence of clinicopathological correlations, the oral lichenoid tissue reaction associated with dental amalgam restorations may also be mistaken for oral lichen planus (OLP) during histopathological examination of biopsy material [7].

OLLs, associated with mostly amalgam restorations, have been attributed to the sensitivity response resulting in immune-mediated damage of the basal epithelial keratinocytes [8]. In addition, some studies have suggested that complete removal of amalgam restorations is required to facilitate clinical healing and histopathological resolution or regression of OLLs as well as diminishing the negative effects of the amalgam materials on the affected patients [9, 10]. Therefore, the differential diagnosis of OLLs should be made on the basis of past medical history, complete mucocutaneous examination, and specific diagnostic tests (i.e., DIF, IIF, and cutaneous patch testing) in addition to clinical and histopathological examination [7].

Extensive dental amalgam restorations can be replaced with a range of restoration materials, including composite resin, acrylic, glass ionomer, gold, metal-bound porcelain, and porcelain [5, 6, 9, 11]. The skin patch test appears to be helpful to the clinician in the determination of suitable replacement material to which the patient lacks a positive reaction [8]. Nevertheless, regardless of the patch test positivity (against any of the amalgam components), complete or marked clinical healing has been reported for OLLs showing close topographical relationships with amalgam restorations [9–13]. For this reason, some studies considered a positive patch test not an actual or independent predictor of improvement of the OLLs following amalgam replacement [8]. However, if the OLL is suspected to be related to amalgam restoration, to completely remove the negative (i.e., toxic, irritant, and allergic) effects of amalgam, precise selection of a replacement material is vital. In recent studies, as adverse reactions associated with the listed materials in patch test results have been disregarded and amalgam restorations have been replaced with primarily composite resin and metal-bound porcelain, which can still result in the formation of OLLs in the oral mucosa, complete healing rates have varied [9, 14]. The healing rate variation undoubtedly is not only associated with material selection, but it is mainly driven by the underlying pathologic condition.

Therefore, the current study aimed to investigate the applicability of feldspathic ceramic as a replacement restoration material for a group of patients with OLLs whose lesions are suspected to be related to amalgam restorations.

## 2. Materials and Methods

This study evaluated twenty-four consecutive patients (seventeen women, seven men; mean age: 45 years; range: 24 to 65 years) with OLLs topographically related to amalgam restorations who were referred to the Department of Oral Surgery and Oral Medicine, Faculty of Dentistry at Istanbul University in Istanbul, Turkey, between May 2007 and February 2008. The clinical criteria for inclusion in this study were the presence of lace-like, white, slightly elevated, reticular, papular, plaque-like, erythematous, erosive, vesiculated, and ulcerative lesions for 3 months or more. To create a specific study group which only consists of patients with OLLs whose lesions are suspected to be associated with their amalgam restorations, we excluded all other patients with OLLs triggered by drugs or those resulting from manifestations of several other diseases, including graft-versus-host disease or lupus erythematous. The latter has been achieved by obtaining the detailed past medical history. On the other hand, prior to the enrollment of participants, a systematic microbiological investigation to exclude any Candida infection was also undertaken. Similarly, detailed blood workup was obtained to rule out hematologic disorders that could present with OLL.

The lesions were biopsied for histopathological examination and underwent epicutaneous patch tests for a series of dental materials (Chemotechnique Diagnostic, Malmo, Sweden). All participants in this study signed consent forms that were approved by the Ethics Committee of Istanbul University, Istanbul Faculty of Medicine, which also approved the study protocol (Project number: 2007/766). Photographs were taken to document the healing process after treatment.

Dental amalgam restoration removal was offered to all 24 initially recruited patients. Sixteen patients agreed to the replacement of their amalgam restorations with feldspathic ceramic inlay-onlay restorations. A rubber dam, high-speed suction, and copious water coolant were used to remove the amalgam restorations to eliminate the negative effects associated with mercury vapor and the risk of exacerbation. After the removal of the amalgam restorations, cavity preparations were completed using an inlay preparation set (4261.314, Komet Dental, Lemgo, Germany), adhering to the general rules of cavity design. Impressions were taken using a vinyl polysiloxane impression material (Express 2 Penta H Universal Quick and Express 2 Light Body Standard Quick, 3M ESPE AG, Seefeld, Germany) using a 1-step impression technique. All inlays and onlays were fabricated from feldspathic ceramic blocks (VITABLOCS Mark II, VITA Zahnfabrik, Bad Sæckingen, Germany) using CAD/CAM technology (CEREC InLab, Sirona Dental, Salzburg, Austria) according to the manufacturer 's instructions. The inlays and onlays were luted with resin-modified glass ionomer cement (GC Fuji Plus, GC Corporation, Tokyo, Japan) on the same day.

We did not prescribe any medication before or after the replacement of the amalgam or in the follow-up period, by virtue of evaluating the healing of OLL just with only one variant which was the feldspathic ceramic. The follow-up examination periods varied between 3 months and 5 years after the replacements. At each follow-up, a clinical examination was performed, and lesion healing/occurrence of new lesions, presence of fractures/chipping, and marginal adaptation of the restorations were evaluated. Study results were analyzed using the chi-square test of independence (x^2^).

## 3. Results 

At the time of recruitment of study participants, 88% of the 24 patients complained of discomfort in the oral mucosa. Sore mouth was the most frequent symptom and was reported in 17 patients (71%). For 8 patients (33%), the complaints were suspected to be related to their amalgam fillings because symptom onset occurred and lesions appeared at the time when the restorations were placed. Eighteen (75%) of the 24 patients had lesions that were limited to areas with direct contact to the amalgam restorations, whereas the remaining 6 patients (25%) had lesions exceeding the contact zone. The most affected area was the buccal mucosa (100%).

All biopsy specimens revealed lichenoid tissue reaction (lichenoid stomatitis). Subsequently, the patients were clinically and histopathologically categorized according to Van der Meij and Van der Waal's modified WHO diagnostic criteria [15]. According to these criteria, 13 patients (54%) fell into the OLL category, whereas the remaining 11 patients (46%) were categorized as OLP. No evidence of dysplastic change was noted in any specimen.

**Figure 1:**
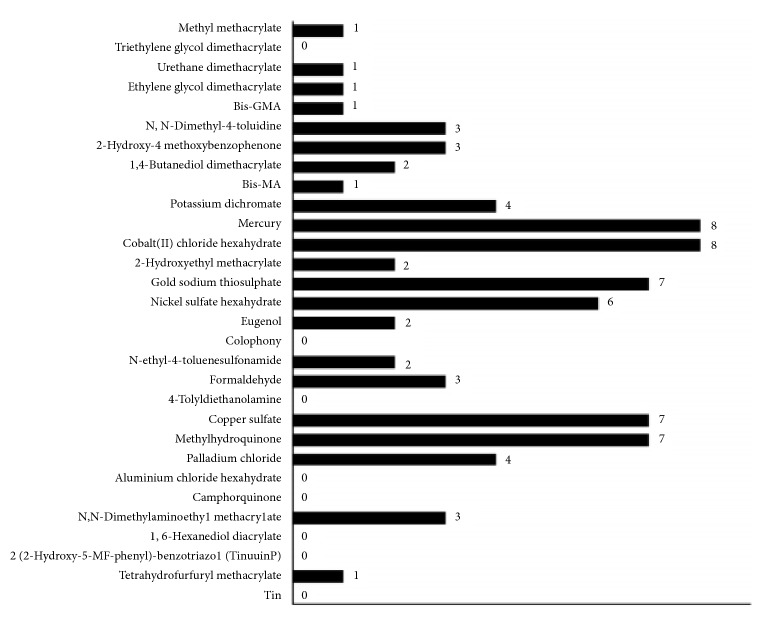
Patients patch test results.

According to patch test results, 22 of the patients (92%) showed sensitization to at least one dental material. The allergens that most commonly elicited a positive reaction were mercury (33%) and cobalt (II) chloride hexahydrate (33%), followed by copper sulfate (30%), gold sodium thiosulfate (30%), methylhydroquinone (30%), and nickel sulfate hexahydrate (25%). Thirteen of the patch-positive patients (59%) showed positive reactions against at least one amalgam component, and 7 of the patch-positive patients (32%) showed positive reactions to at least one composite resin component (Figure 1).

Dental amalgam restoration removal was offered to all 24 initially included patients. Sixteen of the patients accepted the replacement. Therefore, our study group was reduced to 16 patients (67%), 12 of whom were diagnosed with OLLs and 4 of whom were diagnosed with OLP. We replaced the dental amalgam restorations in these 16 patients (all of whom consented to the replacement) with feldspathic ceramic inlay-onlay restorations: 30 ceramic inlay-onlay restorations were placed in the patient group. After 3 months of clinical follow-up and replacement of the amalgam restorations, all of the patients in the study, with the exception of 1 patient with OLP, showed improvement. During the follow-up period, the majority of patients experienced symptom disappearance within weeks of the removal of their amalgam restorations. Furthermore, 10 of the 16 patients (63%) showed complete healing, 5 of the patients (31%) showed marked healing, and the remaining patient (6%) showed no improvement.

Of the 12 patients with OLLs in our study group, 10 patients showed complete healing following the complete replacement of their amalgam restorations with feldspathic ceramic inlay-onlay restorations; all of these patients originally had lesions that were in only close contact with their amalgam restorations. The remaining 2 patients with OLLs showed marked, but incomplete, healing; these patients had lesions that exceeded contact with their amalgam restorations (Table 1).

**Figure 2:**
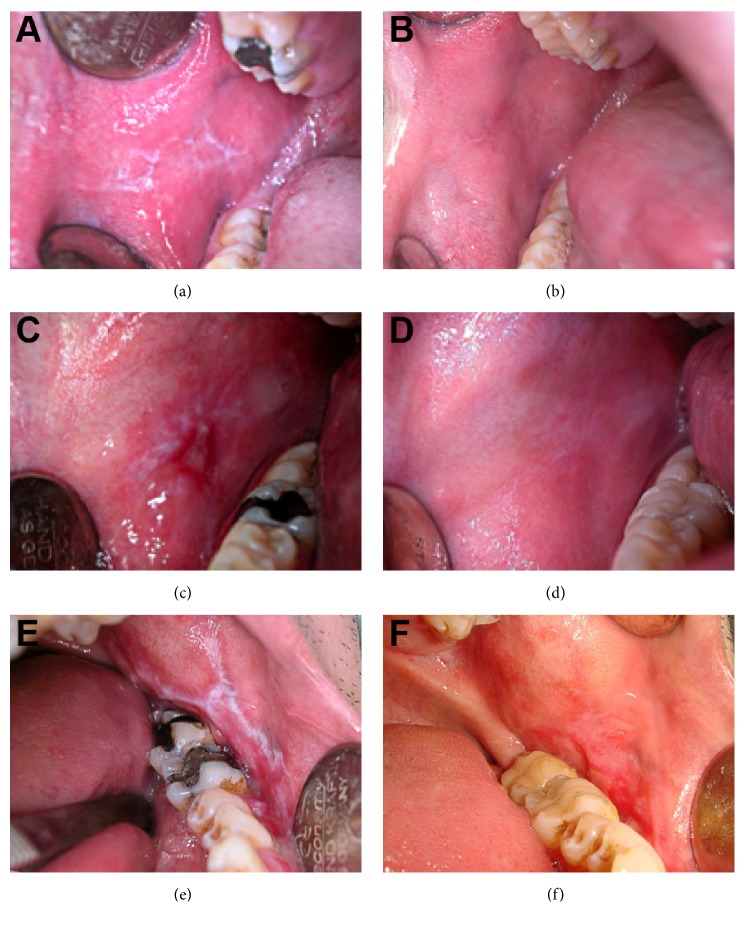
Intraoral views of selected patients before and after treatment. (a), (c), (e) Prior to the replacement with feldspathic inlay-onlay restorations, lichenoid lesions were characterized by white striations. (b), (d), (f) After the replacement with feldspathic inlay-onlay restorations, the lichenoid lesions healed.

Of the 4 patients with OLP in our study group, 3 of the patients showed marked but incomplete healing, and the remaining patient showed no improvement following the complete replacement of their amalgam restorations with feldspathic ceramic inlay-onlay restorations. Our observations, in combination with the evaluated topographical relationships, indicated that complete replacement of amalgam restorations in patients with OLLs resulted in complete healing only when these patients had lesions that were in only close contact to their amalgam restorations (Figure 2). This conclusion was supported by statistical analysis using the chi-square test of independence. According to the statistical data, P<0.05 was considered significant, and healing results (complete/marked healing) were dependent on the topographical relationships that existed between lesions and amalgam restorations (only close contact/exceeding contact with lesions) (x^2^ =5.3, P=0.02).

There was no recurrence of lesions within the observation period, which spanned from 3 months to 5 years. No malignant transformations of OLP or OLL were observed in this study. Of the 30 feldspathic ceramic inlay-onlay restorations that were placed in the 16 patients, 3 failures were found: 1 was caused by ceramic fractures that occurred during the first year of replacement, 1 was caused by tooth fractures that occurred during the third year of replacement, and 1 was caused by secondary caries that occurred during the third year of replacement.

## 4. Discussion

This study attempted to evaluate the success rate of using feldspathic ceramic inlay- onlay restorations as a replacement restoration material and the effect of these replacement restorations on the healing rates of OLLs. To accomplish this task, the impact of the topographical relationship and the healing rate between the lesions and restoration materials were assessed.

Various studies have reported a wide range of oral lesion healing rates (ranging from 37.5% to 100%) following the removal of amalgam restorations [16, 17]. The variation in the healing rates stems from the use of different selection criteria (e.g., replacement restoration material, patch test results, clinical and histopathological diagnosis, and topographic relationship between lesions and amalgams) to determine patient eligibility for amalgam filling replacement. In addition, heterogeneous criteria have been used to define clinical healing in the literature, and a wide range of replacement materials have been used [18]. Lichenoid contact reactions can occur in close proximity to sites of composite resins and various metals, such as gold, palladium, nickel, chrome, and cobalt, and these have been considered to induce the formation of OLLs [19–22].

Regarding replacement restoration materials, in one study, amalgam restorations were replaced with composite, gold, metal-bound porcelain, acrylic, and porcelain, and the clinical healing rate was noted as 69%. The referenced study highlighted that when metal-bound porcelain was used, healing was generally not observed, possibly because of reactions that formed between new tissues and the metal-bound porcelain [10]. In an another study, the amalgam restorations in a patch-positive study group were replaced with composite, glass ionomer, ceramic bonded to precious metal crowns, and gold crowns. The healing rate was 92% in that series. Although that study did not specify a favored replacement material, it suggested the use of inert materials as replacement materials rather than composite fillings. This conclusion was made by considering possible changes that occurred in patient sensitivity to materials included in patch tests because of increased mucosa permeability resulting from the presence of erosive lesions, which could facilitate sensitization to other materials. The conclusion was further supported by the relapse of a lesion in one patient following the replacement of their amalgam restorations with composite fillings based on a negative patch result for Bis-GMA [6]. In a subsequent series, regardless of the status of the patch test, the healing rate was 83% in patients with OLL who underwent amalgam restorations [9]. OLLs can heal when using glass ionomer as a replacement material [6, 23]; however, the leading cause of failure of glass ionomer restorations has been the development of secondary caries [24].

As mentioned above, although previous studies have generally indicated the benefits of the complete removal of amalgam restorations to facilitate the healing of OLLs, based on the varying healing rates that have been observed thus far, no specific restoration material type has yet been suggested as ideal. Since feldspathic ceramic is biocompatible and has not been associated with adverse reactions in the literature, we used it as a replacement restoration material in the current study. Our patients' inlay restorations were cemented using resin-modified glass ionomer cement. It is well known that resin cements show higher bond strengths in comparison with glass ionomer cements or resin-modified glass ionomer cements [25]. Nonetheless, we chose not to use a self-adhesive or self-etch resin cement to keep the resin material content needed for our restoration procedure at a minimum level. Another option is conventional glass ionomer cement; however, resin-modified glass ionomer cements provide stronger enamel bond strength than glass ionomer cements [26]. One study showed that there was no significant difference in durability between resin-modified glass ionomer cement and self-cured resin composite cement when used as luting agents [27]. Regarding the patch test results, we noted that previously reported healing rates associated with amalgam restoration removal are contradictory [10, 28, 29]. Such variations may be due to differences in test methodology which can lead to false-positive or false-negative results [20]. We believe that the patch test is an avoidable procedure during the amalgam restoration removal process. In addition, it should be noted that feldspathic ceramic is not included as a dental series patch test compound. Regarding clinical and histopathological diagnostic considerations, changing a dental restoration material is typically not recommended for the treatment of patients with OLP resulting from unknown causes-etiologies [6, 14, 23]. In the current study, of the 4 enrolled patients with OLP whose amalgam restorations were replaced with feldspathic inlay-onlay ceramic restorations, 3 of them showed only marked healing, and the remaining patient did not show any improvement. While the possibility of amalgam compounds triggering an immunologic response that leads to the development of OLP in the absence of cutaneous disease cannot be excluded, our response rates in 3 of the 4 enrolled OLP cases also raised concerns regarding the accuracy of Van der Meij and Van der Waal's modified WHO diagnostic criteria to distinguish between OLL and OLP. Our observations underscore that, in the absence of clinicopathological correlations, the lichenoid tissue reaction associated with dental amalgam restorations may also be mistaken for OLP during histopathological examination of biopsy material. Therefore, the use of “oral lichenoid tissue reaction or lichenoid stomatitis” in the histopathological diagnosis seems to be much more appropriate for pathologists when patients lack a complete clinical evaluation.

Regarding the topographic relationships that exist between lesions and amalgams, earlier studies have noted that these relationships are closely associated with healing results [11]. In the present study, complete healing was seen in patients with OLLs whose amalgam restorations were in close contact with their lesions after their amalgam restorations were replaced with feldspathic inlay-onlay ceramic restorations. Therefore, our hypothesis was confirmed.

Based on our observations, our recommendation is that dentists may present feldspathic ceramic as a restoration replacement material option rather than composite resin or glass ionomers to their patients with OLLs. Our study was limited to 16 patients, so even though all the patients with OLL whose lesions were in only close contact with their amalgam restorations showed complete healing, larger cohorts must be examined in future studies to compare the cost and benefit of these replacement materials before recommending feldspathic ceramic as the first-line restoration replacement material in OLL patients. This will create specific treatment protocols that enable dentists to utilize appropriate measures upon encountering similar cases.

## 5. Conclusion

This study underscores the impact of appropriate selection of restoration replacement materials in patients with amalgam-driven oral lichenoid tissue reactions. Our results indicate that feldspathic ceramic inlay-onlay restorations can be safely used as a replacement dental restoration material for patients with OLLs to diminish adverse reactions to amalgam restorations.

## Figures and Tables

**Table 1:** Clinical features of the study group and healing results.

	**Complete healing (10)**	**Marked healing (5)**	**No improvement (1)**
Clinical appearance			
Patches, plaques or reticular	10	5	1
Erosive or atrophic	3	2	1
Distribution of lesions			
Buccal mucosa			
Bilateral	3	4	1
Unilateral	7	1	0
Lateral borders of tongue	1	1	0
Diagnosis			
OLL	10	2	0
OLP	0	3	1
Topographical relationship between lesions and restorations			
Only close contact OLL	10	0	0
Exceeding contact OLL	0	2	0
Only close contact OLP	0	2	0
Exceeding contact OLP	0	1	1
Patch test result details			
Patch negative	1	0	1
Patch positive at least one dental material	9	5	0
Patch test response at least one component of amalgam restoration	6	2	0
Patch test response at least one component of composite restoration	2	2	0
Patch test response at least one dental alloy	5	2	0

## Data Availability

The data used to support the findings of this study are included within the article.
